# GFP Fusion to the N-Terminus of MotB Affects the Proton Channel Activity of the Bacterial Flagellar Motor in *Salmonella*

**DOI:** 10.3390/biom10091255

**Published:** 2020-08-29

**Authors:** Yusuke V. Morimoto, Keiichi Namba, Tohru Minamino

**Affiliations:** 1 Department of Physics and Information Technology, Faculty of Computer Science and Systems Engineering, Kyushu Institute of Technology, 680-4 Kawazu, Iizuka, Fukuoka 820-8502, Japan; 2 Graduate School of Frontier Biosciences, Osaka University, 1-3 Yamadaoka, Suita, Osaka 565-0871, Japan; keiichi@fbs.osaka-u.ac.jp (K.N.); tohru@fbs.osaka-u.ac.jp (T.M.); 3 RIKEN Spring-8 Center & Center for Biosystems Dynamics Research (BDR), 1-3 Yamadaoka, Suita, Osaka 565-0871, Japan; 4 JEOL YOKOGUSHI Research Alliance Laboratories, Osaka University, 1-3 Yamadaoka, Suita, Osaka 565-0871, Japan

**Keywords:** bacterial flagellar motor, proton motive force, ion channel, torque generation, fluorescent protein

## Abstract

The bacterial flagellar motor converts the energy of proton flow through the MotA/MotB complex into mechanical works required for motor rotation. The rotational force is generated by electrostatic interactions between the stator protein MotA and the rotor protein FliG. The Arg-90 and Glu-98 from MotA interact with Asp-289 and Arg-281 of FliG, respectively. An increase in the expression level of the wild-type MotA/MotB complex inhibits motility of the *gfp-motB* *fliG(R281V)* mutant but not the *fliG(R281V)* mutant, suggesting that the MotA/GFP-MotB complex cannot work together with wild-type MotA/MotB in the presence of the *fliG(R281V)* mutation. However, it remains unknown why. Here, we investigated the effect of the GFP fusion to MotB at its N-terminus on the MotA/MotB function. Over-expression of wild-type MotA/MotB significantly reduced the growth rate of the *gfp-motB* *fliG(R281V)* mutant. The over-expression of the MotA/GFP-MotB complex caused an excessive proton leakage through its proton channel, thereby inhibiting cell growth. These results suggest that the GFP tag on the MotB N-terminus affects well-regulated proton translocation through the MotA/MotB proton channel. Therefore, we propose that the N-terminal cytoplasmic tail of MotB couples the gating of the proton channel with the MotA–FliG interaction responsible for torque generation.

## 1. Introduction

Many bacteria are propelled by rotating flagella to swim in liquid environments. The basal body is located at the base of the flagellar filament acting as a helical propeller and works as a rotary motor powered by the electrochemical potential difference of cations, such as proton and sodium ion, across the membrane that translocate those cations through the transmembrane channel of the stator complex associated around the rotor [1,2].

The proton-driven flagellar motor of *Salmonella enterica* generates the rotational force through processive interactions between the rotor and multiple stator units [3,4,5,6,7]. A ring-like structure, the basal body MS–C ring complex, functions as a bi-directional rotor, and the switch proteins FliG, FliM, and FliN form the C ring just below the MS ring formed by a transmembrane protein, FliF [8]. The C ring is a switching device that allows the *Salmonella* motor to spin counterclockwise (CCW) and clockwise (CW) [9]. The stator complex is composed of two transmembrane proteins, MotA and MotB, and acts as a transmembrane proton channel that couples the proton flow though the channel with torque generation [10,11,12]. At least 11 stator units can associate around the rotor, but they show rapid exchanges between rotor-associated and freely diffusing forms during motor rotation [13,14]. The flagellar motor autonomously controls the number of functional stator units around the rotor in response to changes in the environment [15].

MotA has four transmembrane (TM) helices (TM1–TM4), two short periplasmic loops, and a relatively large cytoplasmic loop (MotA_C_) between TM2 and TM3 and the C-terminal cytoplasmic tail. MotA_C_ contains highly conserved charged residues, Arg-90 and Glu-98 [16,17]. The motility defect of the *motA(R90E)* and *motA(E98K)* mutants are partially restored by the *fliG(D289K)* and *fliG(R281V)* mutations, respectively, suggesting that electrostatic interactions between Arg-90 of MotA and Asp-289 of FliG and between Glu-98 of MotA and Arg-281 of FliG are responsible for flagellar motor rotation [17].

**Figure 1:**
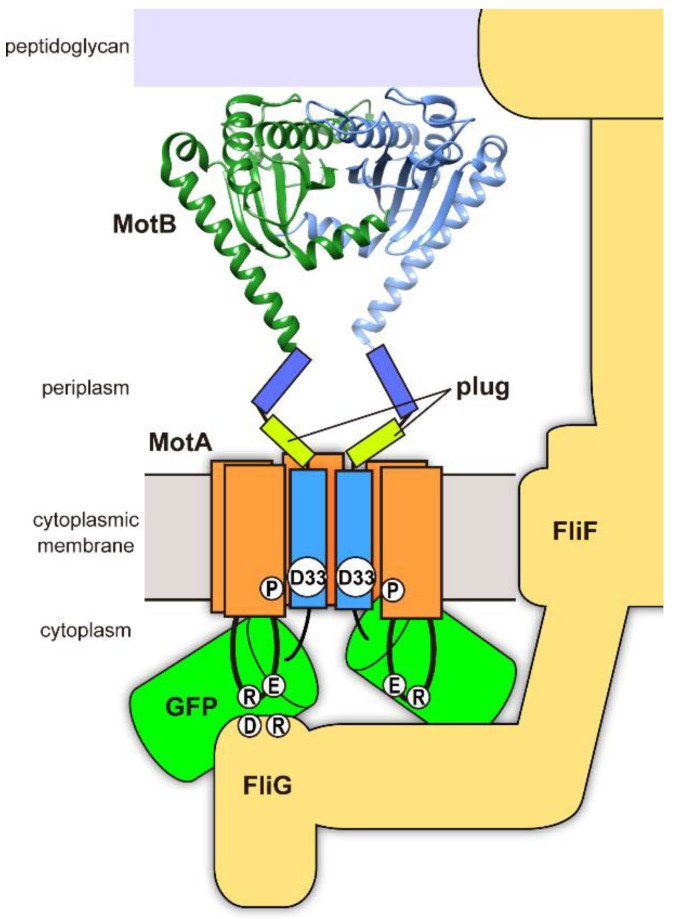
Schematic illustration of the MotA/GFP-MotB complex assembled into the flagellar motor. The N-terminus of MotB faces the cytoplasmic side. MotB-Asp33 (D33) and MotA-P173 (P) residues are located in the proton pathway and play important roles in the energy coupling mechanism of the flagellar motor. Highly charged Arg-90 (R) and Glu-98 (E) residues in the cytoplasmic loop of MotA interact with Asp-289 (D) and Arg-281 (R) of FliG. A plug segment of MotB (plug) suppresses proton leakage through the MotA/MotB complex. The ribbon diagram shows the crystal structure of the PGB domain of MotB (PDB ID: 2ZVY) forming a dimer; its dimerization is critical for the MotB function.

The TM helix of MotB (MotB-TM) form a proton channel along with the TM3 and TM4 helices of MotA. The highly conserved Asp-33 residue of *Salmonella* MotB, which is located in MotB-TM, is the most important residue involved in the proton influx through the MotA/MotB proton channel complex, and the *motB(D33N)* mutation destroys the proton channel activity of the MotA/MotB complex, thereby conferring a loss-of-motility phenotype [18,19,20,21]. The N-terminal tail of MotB (MotB_NCT_) exists in the cytoplasm, and the large C-terminal domain containing a peptidoglycan binding (PGB) motif (MotB_PGB_) is located in the periplasm (Figure 1) [22,23,24]. A flexible linker connecting MotB-TM and MotB_PGB_ contains a plug segment that binds to the proton channel to suppress premature proton translocation through the MotA/MotB proton channel until the MotA/MotB complex becomes an active stator unit around the rotor [25,26]. This flexible linker also suppresses the peptidoglycan binding activity of the MotA/MotB complex until the MotA/MotB complex encounters the rotor, and the interactions between MotA and FliG are postulated to trigger a structural transition of the N-terminal portion of MotB_PGB_ from a compact to an extended conformation, allowing MotB_PGB_ to reach the peptidoglycan (PG) layer for binding [24,27,28]. MotB_NCT_ is critical for the MotB function [29,30], although its role in flagellar motor rotation remains unknown.

Live cell imaging techniques using a fluorescent protein are widely used to elucidate the rotation mechanism of the flagellar motor [13,31,32,33,34,35,36]. A fusion of a green fluorescent protein (GFP) to the N-terminus of MotB does not affect the MotB function much, although the cell motility is not at the wild-type level. Using this functional GFP-MotB fusion, the assembly mechanism of the MotA/MotB stator complex was extensively analyzed in various genetic backgrounds (Figure 1), and we found that the interaction between Arg-90 of MotA and Asp-289 of FliG is more important for proper positioning of the MotA/MotB complex relative to the rotor whereas the interaction between Glu-98 of MotA and Arg-281 of FliG is more critical for torque generation [13,34,36]. However, the fusion of a fluorescent protein to motor component proteins sometimes affects the motor function significantly depending on the fusion sites of the target proteins. For example, simply changing the type of fluorescent protein fused to the N terminus of MotB changes the frequency of directional switching of the flagellar motor [37].

The *Salmonella gfp-motB fliG(D289K)* mutant is non-motile [36]. In contrast, the *gfp-motB fliG(R281V)* mutant is motile with its average swimming speed being about two-thirds of the *gfp-motB* cells [36]. The expression of wild-type MotA/MotB complex restores the motility of the *gfp-motB fliG(D289K)* mutant to about 70% of that of the *gfp-motB* cells, but inhibits the motility of the *gfp-motB fliG(R281V)* mutant while not affecting the motility of the *fliG(R281V)* mutant in the absence of GFP-tagged MotB [36]. These observations suggest that the MotA/GFP-MotB complex cannot work with the wild-type MotA/MotB complex when the *fliG(R281V)* mutation is present. However, it remains unknown how the fusion of GFP to the N-terminus of MotB affects the motor function.

To clarify this question, we analyzed the multicopy effect of the MotA/GFP-MotB complex on intracellular pH to determine whether the GFP tag affects the proton channel activity of the MotA/MotB complex. We found that the over-expression of the MotA/GFP-MotB complex reduces the intracellular pH, thereby causing a growth defect. We also found that MotB_NCT_ is close to both MotA_C_ and FliG and that a fusion of GFP to the N-terminus of MotB facilitates the MotA/MotB proton channel activity regardless of whether or not the MotA/MotB complex being a functionally active stator unit in the motor.

## 2. Materials and Methods

### 2.1. Bacterial Strains, Plasmids and Media

Bacterial strains and plasmids used in this study are listed in Table 1. To construct a plasmid encoding MotA and GFP-MotB, the *motA* and *gfp-motB* genes were amplified by PCR from the chromosomal DNA of the *Salmonella* YVM003 strain, followed by DNA digestion by restriction enzymes, PstI and HindIII, and finally the insertion of the PCR product into the PstI and HindIII sites of the pBAD24 vector. Procedures for DNA manipulations and DNA sequencing were carried out as described previously [38]. L-broth (LB) and motility medium were prepared as described previously [39,40].

### 2.2. Cell Growth

Overnight cultures of *Salmonella* cells grown at 30 °C in LB containing 100 µg/mL ampicillin were diluted 100-fold into fresh LB containing 100 µg/mL ampicillin and 0.2% (*w*/*v*) arabinose, and the cells were grown at 30 °C for 6 h with shaking. The cell growth was monitored at an optical density of 600 nm (OD_600_) every hour. The growth profiles were measured at least three times.

### 2.3. Measurements of Free-Swimming Speeds of Bacterial Cells

For analyses of swimming speeds and swimming fractions, *Salmonella* cells were observed under a phase contrast microscope (IX73, Olympus, Tokyo, Japan) at room temperature. The swimming speed of individual motile cells was analyzed as described previously [45]. Statistical analyses were performed by two-tailed Student’s *t*-test using Prism 7.0c software (GraphPad, CA, USA).

### 2.4. Intracellular pH Measurement

Intracellular pH of *Salmonella* cells was detected using a pH-sensitive red fluorescent protein, mNectarine [44]. SJW1103 and YVM003 were transformed with the pBAD-mNectarine plasmid and grown overnight in LB containing 0.2% (*w*/*v*) arabinose and 100 µg/mL ampicillin at 30 °C. These overnight cultures were measured using a fluorescence spectrophotometer (RF-5300PC, Shimadzu, Kyoto, Japan) with an excitation wavelength at 540 nm and emission at 575 nm as described previously [46].

## 3. Results

### 3.1. Effect of MotA/MotB Over-Expression on Cell Growth in gfp-motB fliG(R281V) Strain

**Figure 2:**
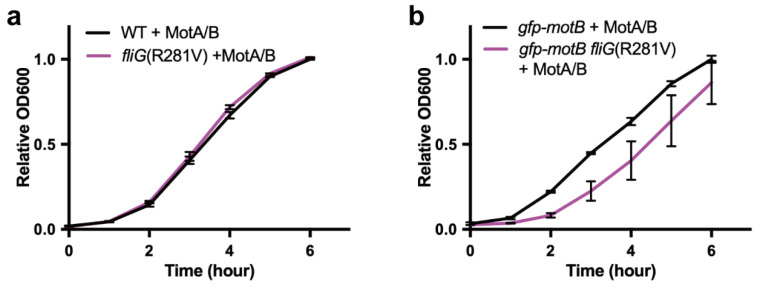
Multicopy effect of the MotA/MotB complex on cell growth. (**a**) Growth curves of the wild-type strain SJW1103 carrying pYC20 (WT + MotA/B, black line) and YVM046 carrying pYC20 (*fliG(R281V)* + MotA/B, magenta line). The values of the optical density at 600 nm were normalized and obtained at 6 h in SJW1103/pYC20. (**b**) Growth curves of the YVM003 carrying pYC20 (*gfp-motB* + MotA/B, black line) and YVM034 carrying pYC20 (*gfp-motB fliG(R281V*) + MotA/B, magenta line). The values of the optical density were normalized and obtained at 6 h in YVM003/pYC20. The cells were grown in LB medium containing 0.2% arabinose and 100 µg/mL ampicillin at 30 °C with shaking. Error bars represent standard deviations.

The proton channel activity of the MotA/MotB complex is suppressed by the plug segment in the MotB linker region when the MotA/MotB complex freely diffuses in the cytoplasmic membrane. Therefore, the expression of the MotA/MotB complex, lacking the plug segment, severely inhibits not only the cell growth, but also motility by reducing the intracellular pH [25,26]. When the intracellular pH decreases, the dissociation rate of protons from the cytoplasmic entrance of the MotA/MotB proton channel into the cytoplasm is reduced significantly, resulting in a slower torque generation cycle of the motor to cause severely impaired motility [47]. These observations lead to a plausible hypothesis that the motility inhibition of the *gfp-motB fliG(R281V)* strain caused by over-expression of the MotA/MotB complex [36] may be a consequence of reduction in intracellular pH caused by undesirable proton flow through the active MotA/MotB proton channel complex in the GFP-MotB/FliG(R281V) motor. To test this hypothesis, we investigated the effect of over-expression of the MotA/MotB complex on the cell growth in the *fliG(R281V)* mutant background. The *Salmonella* wild-type, *fliG(R281V)*, *gfp-motB*, and *gfp-motB fliG(R281V)* strains were transformed with pYC20 encoding MotA and MotB on the pBAD24 vector. These four transformants were grown in LB containing 0.2% arabinose to monitor the cell growth. The over-expression of the MotA/MotB complex did not affect the growth rate of the wild-type and *fliG(R281V)* mutant cells (Figure 2a). Conversely, in the strain expressing GFP-MotB, the *fliG(R281V)* mutation caused a significant delay in the cell growth upon over-expression of the MotA/MotB complex (Figure 2b), indicating that the combination of the GFP tagging to MotB and the *fliG(R281V)* mutation strongly affect the cell growth. This raises the possibility that the interaction of MotB_NCT_ with FliG may also control the gating of the MotA/MotB proton channel during flagellar motor rotation.

### 3.2. Effect of the GFP Tagging on the Proton Channel Activity of the MotA/MotB Complex

**Figure 3:**
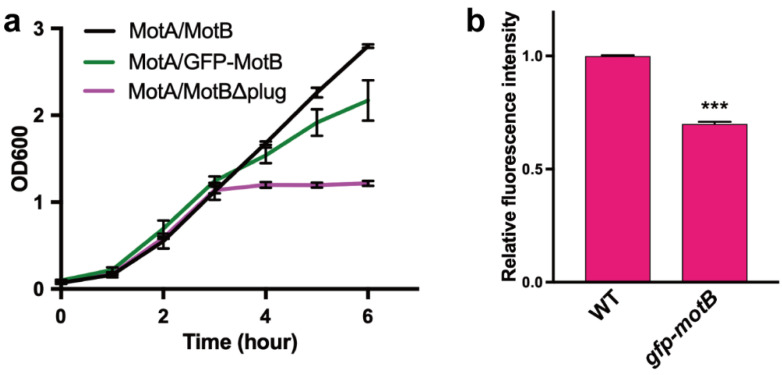
Effect of the GFP tag attached to the N-terminus of MotB on the proton channel activity of the MotA/MotB complex. (**a**) Growth curves of the SJW1368 strain carrying pYC20 (MotA/MotB, black line), pYVM042 (MotA/GFP-MotB, green line) or pYC109 [MotA/MotB (Δplug), magenta line]. The cells were grown in LB at 30 °C with shaking for 3 h, and then arabinose was added to a final concentration of 0.2%. Error bars represent standard deviations. (**b**) Fluorescence intensities of mNectarine in SJW1103 (WT) harboring pBAD-mNectarine and YVM003 (*gfp-motB*) carrying pBAD-mNectarine. The cells were grown overnight in LB at 30 °C with shaking, and then the fluorescence intensities of mNectarine were measured using a fluorescence spectrophotometer. The fluorescence intensities measured in the YVM003 strain were normalized to those of the SJW1103 strain. Statistical analysis was carried out using a two-tailed *t*-test (*** *p* < 0.001). Error bars represent standard deviations.

Next, we investigated whether the GFP tag affects the proton channel activity of the MotA/MotB channel complex incorporated into the flagellar motor in the presence of the *fliG(R281V)* mutation. To test this question, we first analyzed the multicopy effect of the MotA/GFP-MotB on the cell growth in the absence of the flagellar motor to clarify whether the interaction of MotA with FliG(R281V) is responsible for facilitating the proton channel activity of the MotA/GFP-MotB complex in the motor. We over-expressed the MotA/GFP-MotB complex in a flagellar master operon deletion mutant strain, SJW1368, in which no flagellar, motility, and chemotaxis genes are expressed, to measure the proton channel activity of the MotA/GFP-MotB complex by itself. Complexes of wild-type MotA/MotB and MotA/MotB (Δ52–71) lacking the plug segment were used as the negative and positive controls, respectively. Because residues 52–71 of MotB act as the plug that suppresses the proton channel activity of the MotA/MotB complex until the MotA/MotB complex encounters a rotor to become an active stator unit in the flagellar motor (Figure 1), deletion of the plug segment was predicted to result in a marked decrease in the intracellular pH due to massive proton leakage into the cytoplasm, thereby arresting the cell growth [25,26]. Consistently, the expression of MotA/MotB (Δ52–71) drastically interfered with the cell growth (Figure 3a). Although not as much as the plug deletion mutant, a significant growth inhibition was observed in the cells expressing the MotA/GFP-MotB complex compared to the cells expressing the wild-type MotA/MotB complex (Figure 3a), suggesting the possibility that the GFP tag to MotB facilitates excessive proton flow through the MotA/MotB proton channel even in the presence of the plug segment of MotB.

To further clarify the cause of the growth defect, we decided to measure intracellular pH changes by the expression of the MotA/GFP-MotB complex using a pH-sensitive red fluorescent protein, mNectarine. The fluorescence intensity of mNectarine increases significantly with an increase in the surrounding pH from 5.5 to 8.0 [45]. To avoid difficulties in interpreting results due to the over-expression of the membrane protein complex, we expressed mNectarine in the wild-type and *gfp-motB* strains, in which the wild-type MotA/MotB and MotA/GFP-MotB complexes are expressed from their promoter on the chromosomal DNA, and then we measured the fluorescence intensity of mNectarine using a fluorescence spectrophotometer. The fluorescence intensity of mNectarine was significantly lower in the *gfp-motB* cells than the wild-type (Figure 3b), indicating that the GFP tag facilitates the proton channel activity of the MotA/MotB complex even in the presence of the plug segment of MotB, thereby decreasing intracellular pH at the chromosomal expression level. Therefore, we propose that MotB_NCT_ plays an important role in well-coordinated gating of the MotA/MotB proton channel.

### 3.3. Multicopy Effect of Mutant MotA/MotB Complexes on Swimming Motility

**Figure 4:**
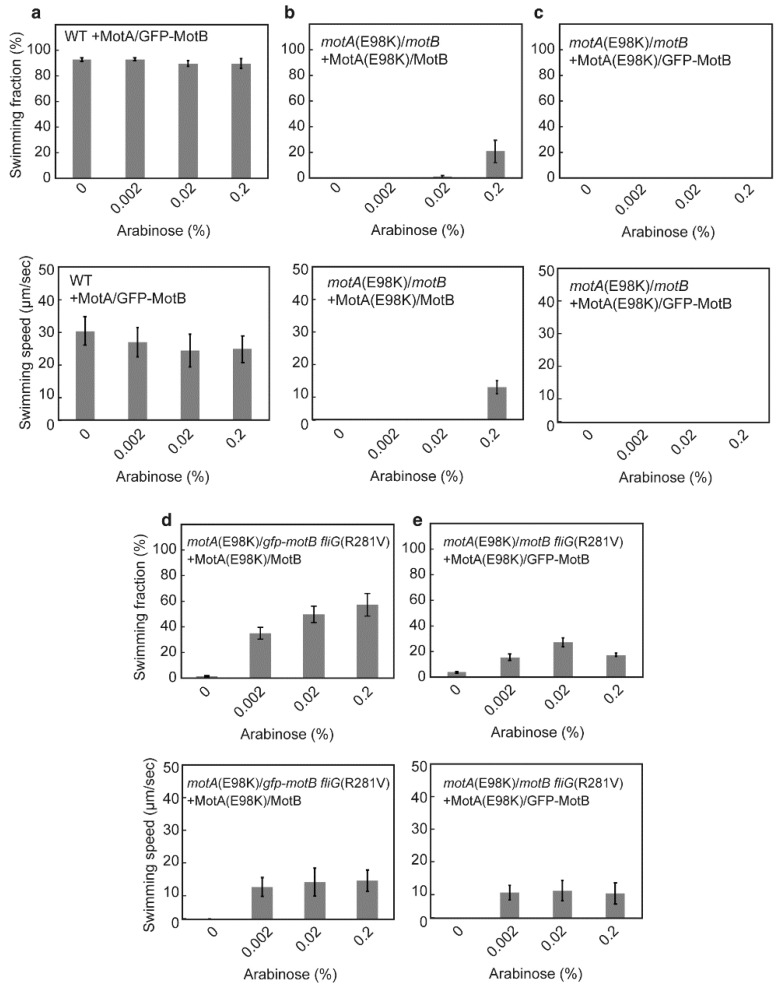
Multicopy effect of MotA/MotB mutants on swimming motility in liquids. The fraction and speed of free-swimming cells were measured for SJW1103 carrying pYVM042 (**a**), YVM47 carrying pYC20(E98K) (**b**), or pYVM042(E98K) (**c**), YVM036 carrying pYC20(E98K) (**d**), and YVM048 carrying pYVM042(E98K) (**e**). Swimming fraction is the fraction of swimming cells. Swimming speed is the average speed of more than 30 cells, and error bars are the standard deviations. If the fraction of motile cells was less than 5% of the total cells, the swimming speed is zero. The cells were incubated at 30 °C for 5 h in LB with 0.2, 0.02, or 0.002% arabinose. Measurements were recorded at around 23 °C.

As described above, a fusion of the GFP tag to the N-terminus of MotB causes excessive proton flow through the MotA/MotB proton channel even in the presence of the plug segment of MotB (Figure 3). Consistently, over-expression of the MotA/GFP-MotB complex reduced the swimming speed of wild-type cells by about 20% (*p* < 0.01) but did not reduce the percentage of motile cells (Figure 4a), possibly due to a decrease in the intracellular pH that reduces the rotational speed of the flagellar motor [47]. It has been shown that the *motA(R90E)* and *motA(E98K)* mutations significantly reduce the efficiency of stator assembly into a motor [34,36] and that an increase in the expression level of the MotA(R90E)/MotB complex restores motility of the *motA(R90E)* mutant to about 60% of the wild-type level [34]. Therefore, we also examined the multicopy effect of the MotA(E98K)/MotB complex on the *motA(E98K)* mutant strain. Only 20% of the cells of the *motA(E98K)* mutant strain showed motility at a markedly reduced level even when the MotA(E98K)/MotB complex was highly expressed by adding 0.2% arabinose (Figure 4b). The over-expression of MotA(E98K)/GFP-MotB complex had no restoration effect on the swimming motility of the *motA(E98K)* mutant (Figure 4c). Because the MotA(E98K)/GFP-MotB complex retains the ability to assemble into the motor to a significant degree [36], this raises the possibility that the MotA/MotB complex with the *motA(E98K)* mutation cannot fully activate its proton channel activity and that the GFP tag to MotB affects such an activation mechanism.

We found that the co-expression of MotB and GFP-MotB has a considerable impact on cell motility and growth in the presence of the *fliG(R281V)* mutation (Figure 2b) [36]. Since it has been reported that the *fliG(R281V)* mutation restores the flagellar motor function of the *motA(E98K)* mutant to a considerable degree [17,36], we next investigated whether this *fliG* suppressor mutation affects the multicopy effect of the MotA(E98K)/MotB complex on the motility of the *motA(E98K) gfp-motB* cells. The fraction of motile cells of the *motA(E98K) fliG(R281V)* strain was less than 5% in the presence or absence of the GFP tag to MotB at the chromosomal expression level of the MotA/MotB stator complex (the left-most bars in the upper panels of Figure 4d,e). However, when the expression level of MotA(E98K)/MotB was increased by adding arabinose, the motile fraction of *motA(E98K) gfp-motB fliG(R281V)* cells increased to about 60% (Figure 4d, upper panel), indicating that the MotA(E98K)/MotB complex is installed into the motor, allowing the *motA(E98K) gfp-motB fliG(R281V)* cells to become motile. In contrast, the average swimming speed of motile cells was unchanged by an increase in the expression level of MotA(E98K)/MotB (Figure 4d, lower panel), suggesting that the interaction between Glu-98 of MotA and Arg-281 of FliG is critical for torque generation as previously proposed [36]. Conversely, MotA(E98K)/GFP-MotB over-expression in the *motA(E98K) fliG(R281V)* mutant cells with 0.02% arabinose increased the motile fraction only to about 30% (*p* < 0.01), and an even higher level of MotA(E98K)/GFP-MotB expression with 0.2% arabinose reduced it again to about 20% (*p* < 0.01; Figure 4e), whereas the average swimming speed did not change over a wide range of the expression level of MotA(E98K)/GFP-MotB. These results suggested that the GFP tag may weaken the interaction between MotA(E98K) and FliG(R281V). Because the GFP tag to MotB increases the proton channel activity of the MotA/MotB complex (Figure 3b), we propose that the interaction between Glu-98 of MotA and Arg-281 of FliG play an important role in the activation mechanism of the MotA/MotB proton channel and that physical communications between MotA_C_ and MotB_NCT_ promote the opening of the cytoplasmic side of the proton channel of the MotA/MotB complex.

## 4. Discussion

Electrostatic interactions between MotA_C_ and FliG are critical not only for efficient stator assembly around the rotor, but also for triggering the detachment of the plug segment from the proton channel to allow MotB_PGB_ to reach and bind to the PG layer, thereby activating the MotA/MotB proton channel for the MotA/MotB complex to become an active stator unit in the motor [24,25,26,28]. The interaction between MotAc and FliG directly transmits a mechanical signal to the MotA/MotB proton channel to regulate its proton channel activity and the affinity of the stator to the rotor to control the number of active stator units around the rotor in response to changes in external loads [48,49,50]. However, its mechanism still remains unknown. Here, we showed that a fusion of GFP to the N-terminus of MotB facilitates the proton channel activity of the MotA/MotB complex (Figure 3). This GFP tag partially inhibits the motility of the *motA(E98K) fliG(R281V)* mutant cells when the MotA(E98K)/GFP-MotB complex is over-expressed (Figure 4e), suggesting that MotB_NCT_ is close to MotA_C_ and FliG. Therefore, we propose that the interaction between MotA_C_ and FliG may induce a conformational change of MotB_NCT_ to open the cytoplasmic side of the proton channel of the MotA/MotB complex for flagellar motor rotation. The *gfp-motB fliG(R281V)* strain is functional, but the over-expression of the MotA/MotB complex causes a non-motile phenotype on this strain, suggesting that the MotA/GFP-MotB complex cannot cooperatively work along with the wild-type in the presence of the *fliG(R281V)* mutation [36]. The *fliG(R281V)* mutation restores the motility of the *motA(E98K)* mutant [17,36]. The *motA(E98K)* mutation does not interfere with the proton channel activity of unplugged MotA/MotB complex while the plugged proton channel cannot be unplugged and activated due to the loss of interaction between MotA and FliG by this MotA mutation [36]. These observations suggest that the MotA/MotB complex with MotA(E98K) mutation cannot activate its proton channel when it encounters FliG in the rotor, whereas the FliG(R281V) mutation allows the proper interaction between MotA_C_ and FliG for the MotA(E98K)/MotB complex to open the channel to become an active stator in the motor. Therefore, we propose that the interaction between FliG and MotA transmits the mechanical signal via MotB_NCT_ to the proton channel, thereby inducing the dissociation of the plug segment from the proton channel to activate the MotA/MotB complex as a stator unit.

## 5. Conclusions

Our results suggest that MotB_NCT_ is close to MotA_C_ and FliG and that a fusion of GFP to the N-terminus of MotB facilitates the MotA/MotB proton channel activity regardless of the MotA/MotB complex becoming a functionally active stator unit in the motor. These observations suggest that the interaction between MotA_C_ and FliG induces a conformational rearrangement of MotB_NCT_, thereby activating the proton channel of the MotA/MotB complex when placed around the rotor.

## Figures and Tables

**Table 1:** Bacterial strains and plasmids.

Strain or Plasmid	Relevant Characteristics	Reference
*Salmonella*		
SJW1103	Wild-type for motility and chemotaxis	[41]
SJW1368	∆*(cheW-flhD)*; master operon mutant	[42]
YVM003	*gfp-motB*	[34]
YVM034	*gfp-motB fliG(R281V)*	[36]
YVM036	*motA(E98K) gfp-motB fliG(R281V)*	[36]
YVM046	*fliG(R281V)*	[36]
YVM047	*motA(E98K)*	[36]
YVM048	*motA(E98K) fliG(R281V)*	[36]
*Plasmid*		
pBAD24	Expression vector	[43]
pYC20	pBAD24/MotA+MotB	[26]
pYC20(E98K)	pBAD24/MotA(E98K)+MotB	[34]
pYC109	pBAD24/MotA+MotB(∆52–71)	[26]
pYVM042	pBAD24/MotA+GFP-MotB	This study
pYVM042(E98K)	pBAD24/MotA(E98K)+GFP-MotB	This study
pBAD-mNectarine	pBAD/His-mNectarine (addgene #21717)	[44]
